# Genetic Liability to Bone Mineral Density and Functional Outcome After Ischemic Stroke

**DOI:** 10.1002/brb3.71068

**Published:** 2025-11-14

**Authors:** Daofeng Fan, Jiaqian Dai, Xingxing Ye, Yinjuan Chen, Yangui Chen, Wenbao Wu

**Affiliations:** ^1^ Department of Neurology Longyan First Hospital Affiliated to Fujian Medical University Longyan Fujian China; ^2^ Department of Otorhinolaryngology Longyan Second Hospital of Fujian Province Longyan Fujian China; ^3^ Department of Acupuncture and Moxibustion Longyan First Hospital Affiliated to Fujian Medical University Longyan Fujian China

**Keywords:** bone mineral density, functional outcome, heel bone density, ischemic stroke, mendelian randomization

## Abstract

**Background and Objectives:**

An increasing amount of research indicates that bone mineral density (BMD) could impact the functional outcome after ischemic stroke (FOAIS). However, the specific connection between BMD and FOAIS is still not clear. Hence, we utilized the Mendelian randomization method to examine the genetic predisposition to BMD in connection with FOAIS.

**Methods:**

Instrumental variables for BMD, heel bone mineral density T score (HBMDTS), and lumbar spine bone mineral density (LSBMD) were identified from genome‐wide association study data of individuals with European ancestry. The study on FOAIS data came from European ancestry patients and was carried out by the Genetics of Ischemic Stroke (IS) Functional Outcome network. The inverse variance weighted method was employed to assess the genetic correlation between BMD and FOAIS. To assess the reliability of the results, sensitivity analyses were performed employing alternative Mendelian randomization techniques, such as the weighted median approach and MR analysis using the Robust Adjusted Profile Score. Furthermore, the intercept from MR‐Egger regression was applied to identify possible pleiotropic effects. The variability among genetic variants was evaluated through I^2^ and Q statistics.

**Results:**

Genetic liability to HBMDTS was associated with poor FOAIS at 3 months (mRS 3–6, IVW OR = 1.30; 95% CI = 1.09–1.54; *p* = 0.003, *p‐Bonferroni* = 0.009). Even after adjusting for relevant factors, significant associations remained (mRS 3–6, IVW OR = 1.33; 95% CI = 1.09–1.63; *p* = 0.006, *p‐Bonferroni* = 0.017). No significant causal effects were observed for total BMD or LSBMD on FOAIS. Sensitivity analyses showed no evidence of pleiotropy or heterogeneity.

**Conclusions:**

Genetic predisposition to HBMDTS was associated with poor FOAIS. This research provides a genetic basis for the association between HBMDTS and poor FOAIS, potentially impacting the clinical significance of recovery for stroke patients.

## Graphical abstract

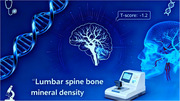

## 1 Introduction

Ischemic stroke (IS) was the predominant type of stroke, representing 62.4% of all stroke occurrences and associated with substantial long‐term neurological dysfunction and elevated mortality rates (Roth et al. 2020, Hankey 2014). Functional outcome after ischemic stroke (FOAIS) is a critical measure of recovery, typically assessed using the modified Rankin Scale (mRS) at three months post‐stroke. Identifying factors that influence FOAIS is essential for developing targeted interventions to improve prognosis and reduce the burden of stroke‐related disability.

Osteoporosis, characterized by reduced bone mineral density (BMD), is a common systemic skeletal disorder affecting approximately 23% of women and 11% of men globally (Salari et al. 2021; Center et al. 2011). BMD is a widely used clinical indicator of bone strength and osteoporosis risk. Notably, heel BMD measured by quantitative ultrasound (QUS) has been validated as a reliable proxy for overall BMD (Taal et al. 1999; Khaw et al. 2004), making it a practical tool for large‐scale genetic studies.

An emerging body of evidence suggests a potential link between BMD and stroke outcomes. Several observational studies have reported associations between low BMD and increased stroke risk (Browner et al. 1993; Myint et al. 2014; Zhou et al. 2015; Mussolino and Armenian 2007; Zhu et al. 2022), while others have indicated that reduced BMD may predict poorer functional recovery after stroke (Nordström et al. 2010; Sohn et al. 2023; Lee et al. 2013; Schnitzer et al. 2012). However, these findings remain inconsistent, and the causal nature of this relationship remains uncertain due to potential confounding factors and reverse causality inherent in observational study designs.

Mendelian randomization (MR) has emerged as a powerful methodological approach that uses genetic variants as instrumental variables to infer causal relationships between exposures and outcomes (Davey Smith and Hemani 2014; Gupta et al. 2017). By leveraging genetic variants randomly assigned at conception, MR minimizes confounding and reverse causation biases that often plague observational studies. This method is particularly suited for investigating the potential causal effect of BMD on FOAIS, as genetic determinants of BMD are established early in life and cannot be modified by subsequent stroke occurrence or recovery processes.

In this study, we conducted a bidirectional two‐sample MR analysis to investigate the causal relationship between genetic liability to BMD (including heel bone mineral density T score (HBMDTS) and lumbar spine bone mineral density (LSBMD)) and FOAIS. Utilizing large‐scale genome‐wide association study (GWAS) summary statistics, we aimed to determine whether genetic predisposition to higher BMD influences functional recovery after ischemic stroke, potentially informing novel therapeutic strategies for stroke rehabilitation.

## 2 Methods

**FIGURE 1:**
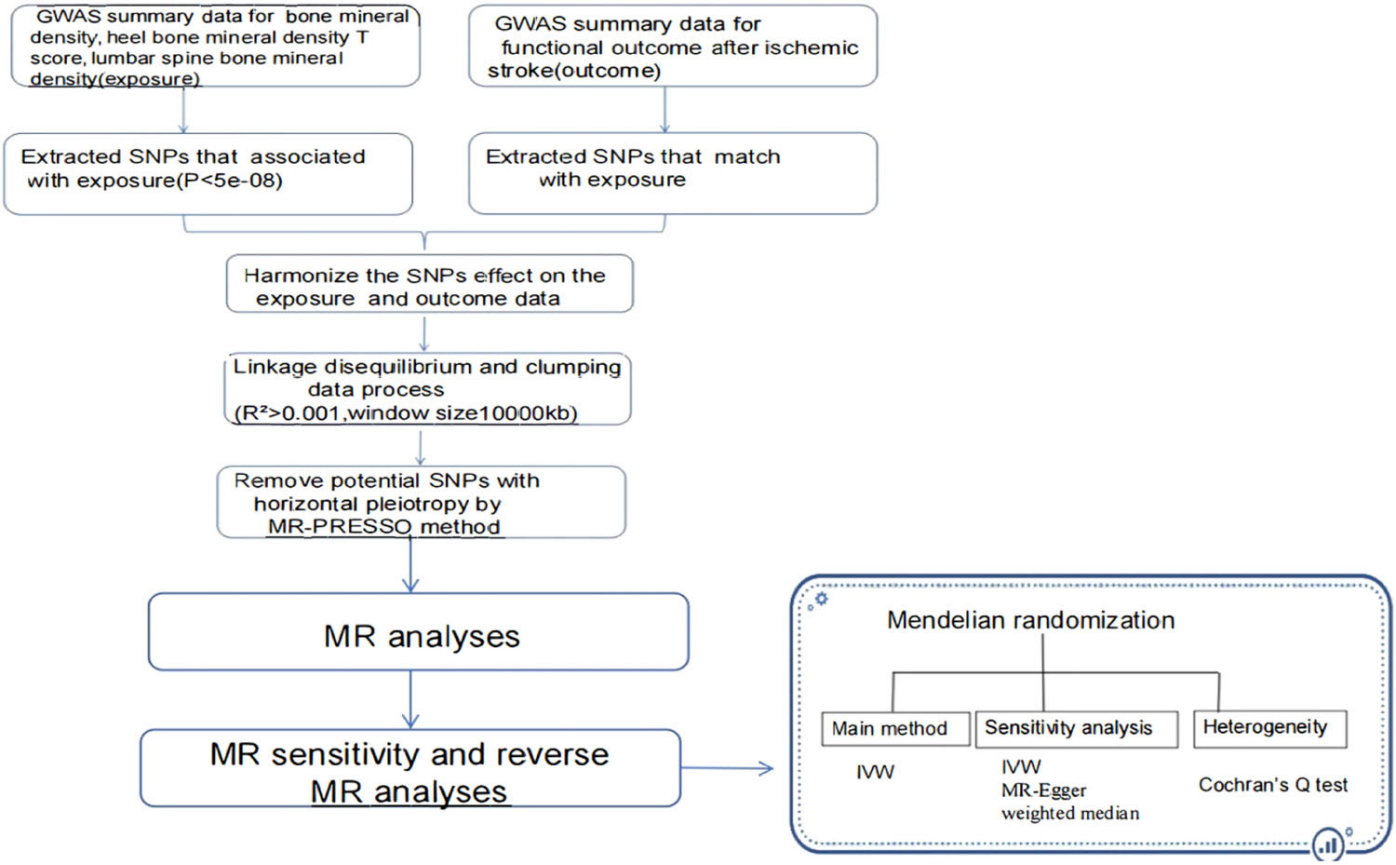
**FIGURE 1** Study design. GWAS, genome‐wide association study; SNPs, single‐nucleotide polymorphisms; MR, Mendel randomized; IVW, inverse variance weighted.

Figure 1 depicts the general layout of the research. We carried out a two‐sample bidirectional MR analysis that utilizes the summary statistics for BMD, HBMDTS, LSBMD, and FOAIS from two distinct, unrelated populations. To minimize bias due to population stratification, our study was confined to individuals of European descent.

## 3 BMD and HBMDTS and LSBMD Data Sources

We acquired GWAS summary data for BMD from the United Kingdom Biobank, which includes 365,403 individuals of European descent (ebi‐a‐GCST90014022) (Mbatchou et al. 2021), and HBMDTS were obtained for 445,855 cases with European ancestry from UK Biobank (ebi‐a‐GCST90025982) (Barton et al. 2021). GWAS summary data for LSBMD (measured at the lumbar spine (L1–L4)) were obtained for 28,498 cases of European ancestry from the UK 10K (ieu‐a‐982) (Yang and Sui 2022). As Table 1 shows the sources of the data used in this study, the following is a description of the various data.

**TABLE 1:** Detailed information on included traits in this study.

Phenotype	Sample size	Ancestry	Significance level	Data Sources	PMID
BMD	365,403	European	5e−8	UK Biobank (ebi‐a‐GCST90014022)	34017140
HBMDTS	445,855	European	5e−8	UK Biobank (ebi‐a‐GCST90025982)	34226706
LSBMD	28,498	European	5e−8	UK 10K (ieu‐a‐982)	26367794
Functional outcomes after ischemic stroke	6,021	European	5e−8	GISCOME ischemic	30796134
Ischemic stroke	40,585	European	5e−8	MEGASTROKE Consortium (ebi‐a‐GCST005838)	29531354

### 3.1 FOAIS Data Sources

FOAIS was assessed utilizing the modified Rankin Scale (mRS) at 3 months post‐stroke (range: 0 ‐ no symptoms; 6 ‐ death). mRS score greater than 2 was considered an unfavorable. The GWAS carried out by the Genetics of Ischemic Stroke Functional Outcome (GISCOME) network provided the genetic estimates for mRS score at 3 months. The GISCOME research utilized a range of statistical models, such as binary comparisons between mRS 0–2 and 3–6, binary comparisons between mRS 0–1 and 2–6, and ordered models for mRS. We applied the GWAS summary data to the adjusted comparison of mRS 0–2 vs 3–6, which reflected stroke severity based on the baseline National Institute of Health stroke scale (NIHSS). Moreover, the GWAS model that utilized dichotomous mRS 0–2 vs 3–6 (*N* = 6,021), adjusted for NIHSS, features a more comprehensive sample size than the model that used dichotomous mRS 0–1 vs 2–6, adjusted for NIHSS (*N* = 4,363). In the GWAS model, 6,021 patients of European descent with IS, with a mRS score ranging between 0 and 6, were analyzed, of which 2,280 had a score of 3 to 6 and 3,741 had a score of 0 to 2. The genetic variations identified by the GWAS were adjusted for several relevant variables, such as age, sex, ancestry, and baseline NIHSS score, and were divided into uncorrected and corrected data. The detailed information of these data were presented in Table 1.

### 3.2 Instrumental Variable Selection

First of all, a threshold of *p* < 5 × 10^−8^ was first used to screen SNPS that showed strong associations with BMD, HBMDTS and LSBMD. Furthermore, in order to account for potential linkage disequilibrium (LD), these SNPs were effectively organized using a 10,000 kb boundary and an *r* ^2^ = 0.001 cutoff. The 10,000 kb distance threshold is commonly used in LD‐based clumping to ensure that selected SNPs are independent and not in LD. This value is based on the typical extent of LD in European populations, where LD rarely extends beyond 10,000 kb. In cases where specific SNPs exceeded the LD threshold of 0.1, the associated SNPs with the most minimal *P*‐values were chosen for subsequent analysis. Then, we use the ensemble database to extract possible traits and to identify SNPs of confounding factors that may affect the FOAIS, and we remove them. The *F*‐value for every instrumental variable needs to be over 10 (Palmer et al. 2012; Levin et al. 2020; Gill et al. 2019). Through the above steps, we screened 481 SNP (BMD), 451 SNP (HBMDTS), 23 SNP (LSBMD), which were correlated with uncorrected FOAIS (UFOAIS), and also screened 478 SNP (BMD), 419 SNP (HBMDTS), 23 SNP (LSBMD) were correlated with corrected FOAIS (CFOAIS) (Supplementary Table S1‐S6). UFOAIS: mRS 0–2 vs. 3–6 without adjustment for age, sex, ancestry, and baseline NIHSS score. CFOAIS: mRS 0–2 vs. 3–6 with adjustment for age, sex, ancestry, and baseline NIHSS score.

### 3.3 Evaluating Genetic Liability Association BMD, HBMDTS, LSBMD With IS Risk

Collider bias is a potential source of bias that may impact the accuracy of MR‐based analyses related to disease prognosis. To minimize this bias, we assessed the genetic liability association between BMD, HBMDTS, LSBMD, and IS. The MEGASTROKE Consortium provided comprehensive summary statistics for IS, a large‐scale study involving 40,585 cases and 406,111 controls of European descent (Table 1).

### 3.4 Statistical Analysis

#### 3.4.1 Estimation of Causal Effect

To assess the associations between BMD, HBMDTS, LSBMD and FOAIS, multiple statistical methods including inverse variance weighted (IVW) (Burgess et al. 2015; Burgess et al. 2013), MR‐Egger regression (Bowden et al. 2015), Simple mode, Weighted mode (Hartwig et al. 2017) and weighted median (Bowden et al. 2016) approaches were used to examine the potential causal relationship. Then apply the same method for reverse MR analysis. Only these five methods come out in the same direction and we consider the results reliable. The consistency in the direction of results obtained by multiple Mendelian randomization methods, especially IVW, weighted median, and MR‐Egger, enhances the reliability of causal inference as it reduces the risk of bias caused by pleiotropy and model misspecification. (Burgess et al. 2013; Bowden et al. 2016). Odds ratios (OR) and 95% confidence intervals (CI) were reported for the effect estimates.

#### 3.4.2 Heterogeneity and Sensitivity Analysis

Cochran's Q test was used to assess heterogeneity, which was evaluated using both the IVW and MR‐Egger methods. A *p*‐value > 0.05 indicates no significant heterogeneity. Heterogeneity is assumed to be absent when the *p*‐value is greater than 0.05 (Burgess et al. 2013). The Egger intercept method is used to test pleiotropy. A p‐value greater than 0.05 indicates that pleiotropy does not exist (Bowden et al. 2015). To ascertain the overall causal effects, we implemented a leave‐one‐out sensitivity analysis technique, systematically removing one SNP at each iteration. If the results remained consistent after removing each SNP, it indicated that the findings were robust and not driven by any individual variant. The method of MR‐PRESSO was utilized to analyze the outliers of instrumental variables with pleiotropic effects, with a significance threshold of *p* < 0.05 (Verbanck et al. 2018). We used Bonferroni's correction to account for multiple testing across three exposures: BMD, HBMDTS, and LSBMD. Therefore, the significance threshold was set at 0.05/3 = 0.0167. The statistical analysis was conducted utilizing the Two Sample MR package (version 0.5.7), Mendelian Randomization (version 1.0), and MR‐PRESSO in R (version 4.3.1).

## 4 Results

### 4.1 Association of BMD With UFOAIS and CFOAIS

**FIGURE 2:**
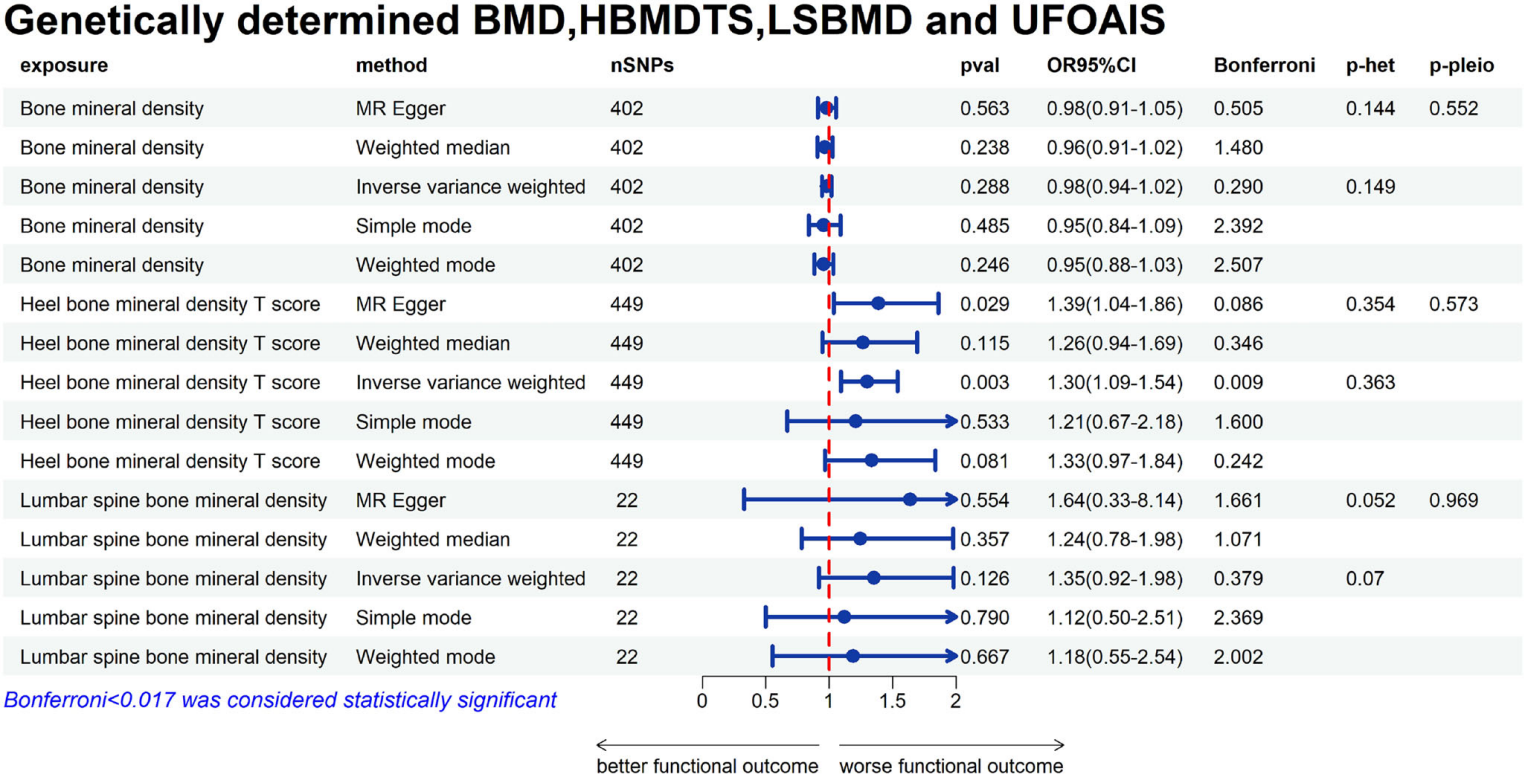
Genetically determined BMD, HBMDTS, LSBMD, and UFOAIS. BMD, bone mineral density; HBDTS, heel bone mineral density T score; LSBMD, lumbar spine bone mineral density; UFOAIS, uncorrected functional outcomes after ischemic stroke; CI, confidence interval; OR, odds ratio; SNPs, single nucleotide polymorphisms.

**FIGURE 3:**
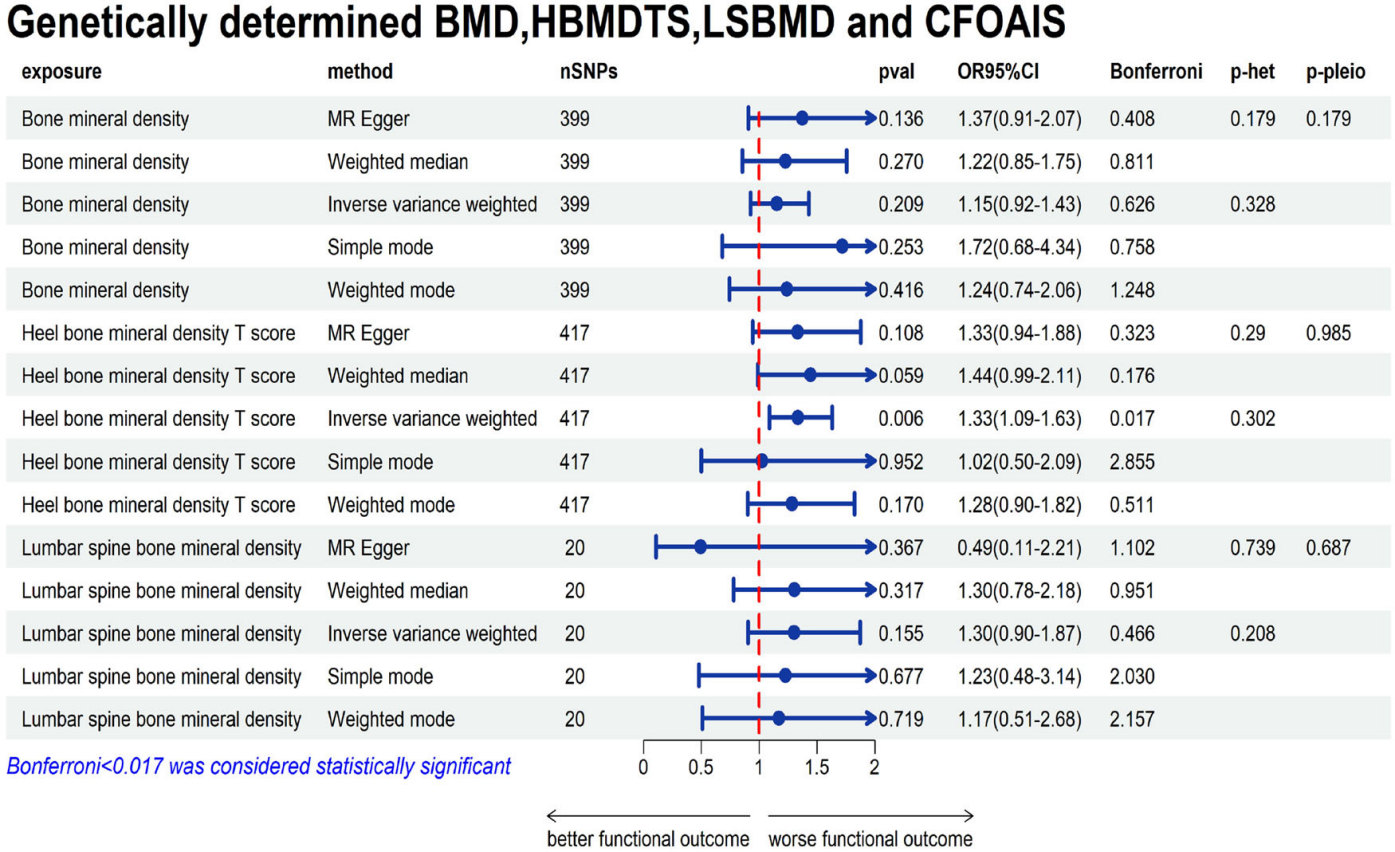
Genetically determined BMD, HBMDTS, LSBMD, and CFOAIS. BMD, bone mineral density; HBMDTS, heel bone mineral density T score; LSBMD, lumbar spine bone mineral density; CFOAIS, corrected functional outcomes after ischemic stroke; CI, confidence interval; OR, odds ratio; SNPs, single nucleotide polymorphisms.

The main analysis did not find a significant correlation between BMD and UFOAIS (mRS 3–6, IVW OR = 0.98; 95% CI = 0.94–1.02; *p* = 0.288), and heterogeneity and horizontal pleiotropy were not present (heterogeneity‐IVW = 0.363, heterogeneity‐MR Egger = 0.354, pleiotropy = 0.573). BMD and CFOAIS did not have a significant association (mRS 3–6, IVW OR = 1.15; 95% CI = 0.92–1.43; *p* = 0.209), and heterogeneity and horizontal pleiotropy were not present (heterogeneity‐IVW = 0.302, heterogeneity‐MR Egger = 0.290, pleiotropy = 0.985). Figures 2 and 3 depict the sensitivity analyses of MR.

### 4.2 Association of HBMDTS With UFOAIS and CFOAIS

**FIGURE 4:**
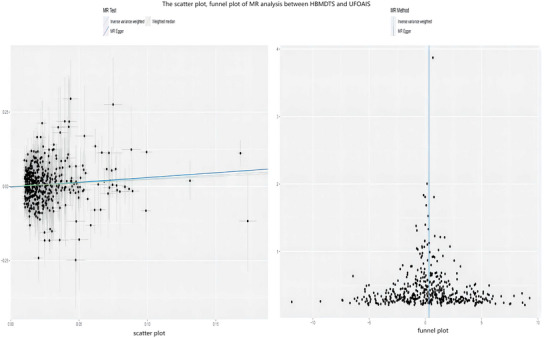
The scatter plot, funnel plot of MR analysis between HBMDTS and UFOAIS. HBMDTS, heel bone mineral density T score; UFOAIS, uncorrected functional outcomes after ischemic stroke.

**FIGURE 5:**
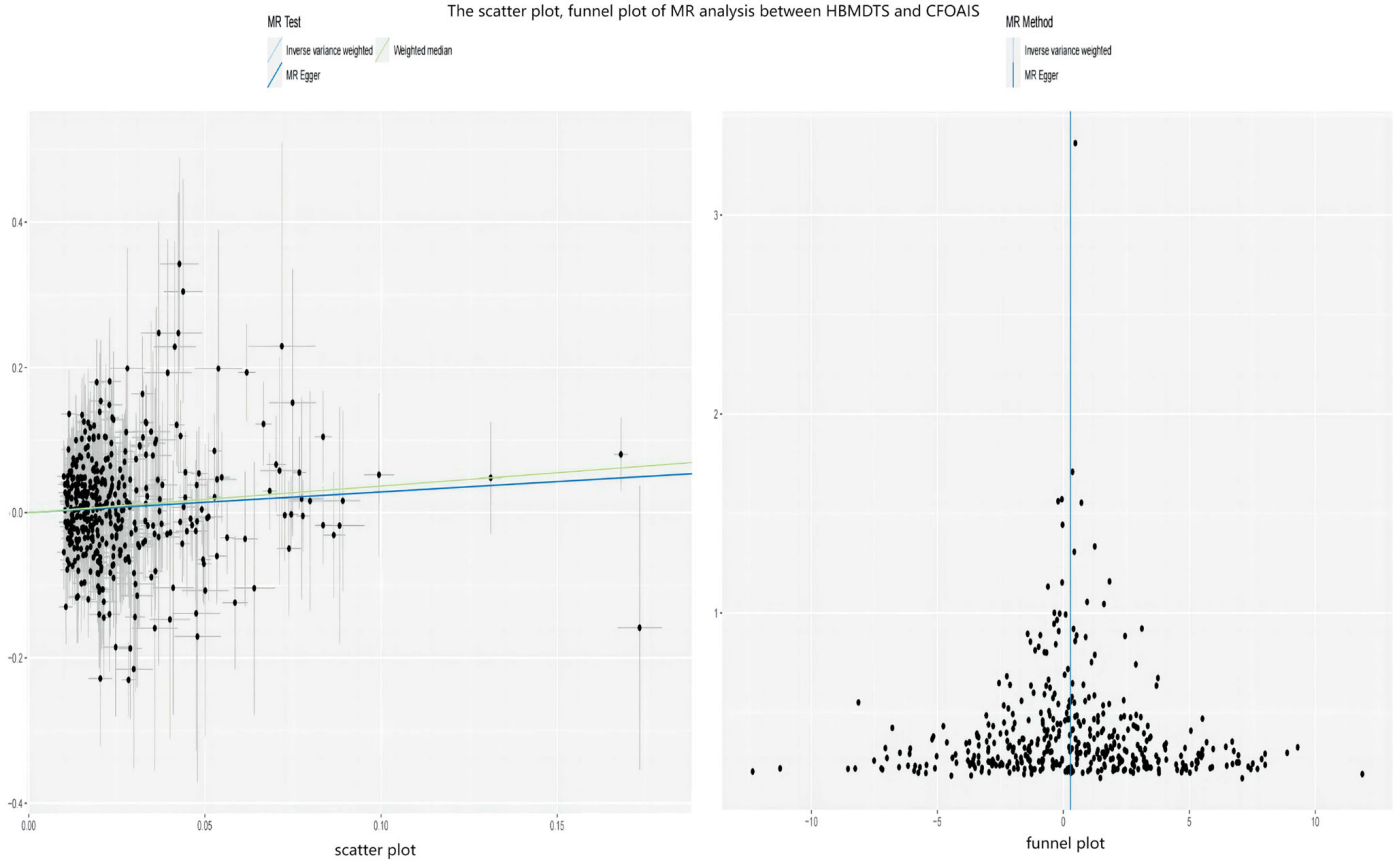
The scatter plot and funnel plot of MR analysis between HBMDTS and CFOAIS. HBMDTS, heel bone mineral density T score; CFOAIS, corrected functional outcomes after ischemic stroke.

A significant association between HBMDTS and UFOAIS was found in the main analysis (mRS 3–6, IVW OR = 1.30; 95% CI = 1.09–1.54; *p* = 0.003, *p‐Bonferroni* = 0.009), and there was no heterogeneity or horizontal pleiotropy (heterogeneity‐IVW = 0.363, heterogeneity‐MR Egger = 0.354, pleiotropy = 0.573) (Figure 2). There was a significant association between HBMDTS and CFOAIS (mRS 3–6, IVW OR = 1.33; 95% CI = 1.09–1.63; *p* = 0.006, *p‐Bonferroni* = 0.017), and there was no heterogeneity or horizontal pleiotropy (heterogeneity‐IVW = 0.302, heterogeneity‐MR Egger = 0.290, pleiotropy = 0.985) (Figure 3). The weighted median and MR‐Egger both demonstrate consistent findings with IVW (Figures 2 and 3). The sensitivity analyses of MR are shown in Figures 2 and 3. The scatter plot and funnel plot of the MR analysis the association of HBMDTS with UFOAIS and CFOAIS are shown in Figures 4 and 5. The leave‐one‐out plot of association of HBMDTS with UFOAIS and CFOAIS MR analyses is shown in Supplementary Figs. S1–S2 online.

### 4.3 Association of LSBMD With UFOAIS and CFOAIS

A significant association between LSBMD and UFOAIS was not found in the main analysis (mRS 3–6, IVW OR = 1.35; 95% CI = 0.92–1.98; *p* = 0.126) and there was no significant correlation with CFOAIS (mRS 3–6, IVW OR = 1.30; 95% CI = 0.90–1.87; *p* = 0.155). Figures 2 and 3 depict the sensitivity analyses of MR.

### 4.4 Association of BMD, HBMDTS, LSBMD With IS

We found a total of 404, 445, and 19 genetic variants, each independently associated with IS, in the BMD, HBMDTS, and LSBMD datasets. We found that BMD (mRS 3–6, IVW OR = 0.979; 95% CI = 0.94–1.02; *p* = 0.288), HBMDTS (mRS 3–6, IVW OR = 0.967; 95% CI = 0.93–1.00; *p* = 0.090), and LSBMD (mRS 3–6, IVW OR = 1.037; 95% CI = 0.97–1.11; *p* = 0.281) were not associated with IS, which suggests that their potential connection with functional outcomes may not be due to the presence of collider bias.

### 4.5 Reverse MR

To establish an association of FOAIS with BMD, HBMDTS, and LSBMD, a reverse MR study is conducted. Our reverse MR results indicate that FOAIS is not associated with BMD, HBMDTS, or LSBMD, and that IS is not associated with any of them (see Supplementary Figure S3 online and Supplementary Table S7).

## 5 Discussion

Our research involved a bidirectional two‐sample MR, which examines the causal relationship association BMD, HBMDTS, LSBMD with FOAIS. This study is the first to assess how genetic susceptibility to HBMDTS affects FOAIS. Our research indicates that HBMDTS is linked to poor functional outcomes in patients who have suffered from IS. However, BMD and LSBMD did not have a causal relationship with FOAIS. The findings do not appear to be driven by collider bias or horizontal pleiotropy.

It is not clear whether BMD and FOAIS are associated in observational studies. A 2‐year follow‐up cohort study on elderly females revealed a notable correlation between decreased BMD and mortality from stroke (Browner et al. 1991). Lee et al. (2013) revel that decreased BMD in the right femur is linked to poor outcome three months following an IS. Nevertheless, a significant correlation between BMD and stroke mortality in both white and black individuals was not observed (Mussolino et al. 2003). Conversely, Sohn et al. (2023) revealed that those who were administered osteoporosis medication demonstrated significantly improved FOAIS, implies that there is a potential link between decreased BMD and poor FOAIS. Our discovery indicates a strong correlation between the genetic predisposition for HBMDTS and FOAIS.

The link between HBMDTS and poor FOAIS is not well understood, and the current analysis does not offer any clarification on this issue. One possible interpretation is that patients with IS due to osteoporosis experience a significant decrease in bone density in the early stages, resulting in discomfort and hindering the patient's capacity to engage in physical activities, ultimately resulting in poor functional recovery. In addition, reduced BMD can lead to an increased risk of fracture, resulting in poor functional recovery (Sànchez‐Riera et al. 2014; Yang et al. 2020). Another potential factor is the possibility that reduced BMD could elevate the occurrence of cerebral small vessel disease, consequently impacting the recuperation of neurological function after IS (Kim et al. 2021).

Even though we have performed a bidirectional MR analysis to eliminate reverse causation, our analysis still has numerous shortcomings. First, while we analyzed the connection between BMD and LSBMD in relation to FOAIS, we were unable to explore the link between femoral neck density and FOAIS due to the lack of relevant GWAS data. Secondly, the female hormone estrogen may play a role in BMD and FOAIS, however, due to the insufficient availability of relevant GWAS data, we cannot determine whether the bias is related to gender. Third, the data do not differentiate between different types of strokes, so we cannot further explore the correlation between BMD and the prognosis of various stroke types. Fourth, these data primarily involve European populations, and additional studies are necessary to confirm whether the results are applicable to other populations. Fifth, the genetic FOAIS are derived from a retrospective analysis with a relatively limited sample size, potentially leading to survival rate and selection bias effects. Therefore, it is important to exercise caution when interpreting the results. In the future, we expect to see more research data being incorporated regarding HBMDTS or BMD and its association with FOAIS. Additionally, we look forward to the execution of comprehensive cohort studies aimed at delving deeper into the causal relationship.

## 6 Conclusion

In summary, the MR analysis provided evidence that genetic predisposition to HBMDTS was associated with poor FOAIS. Future studies need to replicate and confirm our findings in larger samples of patients from different subgroups.

## Author Contributions

**Daofeng Fan**: conceptualization, investigation, writing – original draft. **Jiaqian Dai**: conceptualization, investigation, writing – original draft. **Xingxing Ye**: data curation, formal analysis, writing – review and editing. **Yinjuan Chen**: data curation, formal analysis, writing – review and editing. **Yangui Chen**: data curation, formal analysis, writing – review and editing. **Wenbao Wu**: supervision, project administration, writing – review and editing.

## Funding

This research was financially supported by Fujian Province Natural Science Foundation (Grant No: 2023J011879) and Longyan City Science and Technology Plan Project (Grant No: 2022LYF17042, 2023LYF17043, 2023LYF17044).

## Ethics Statement

The data for this study were retrieved from previously published GWAS. Each institutional review board participating in this research obtained written informed consent from all the participants in their respective studies. This study was carried out in accordance with the Helsinki Declaration revised in 2013.

## Conflicts of Interest

The authors declare no conflicts of interest.

## Peer Review

The peer review history for this article is available at https://doi.org/10.1002/brb3.71068.

## Supporting information




**Supplementary Figure**: brb371068‐sup‐0001‐FigureS1.tif

**Supplementary Figure**: brb371068‐sup‐0002‐FigureS2.tif

**Supplementary Figure**: brb371068‐sup‐0003‐FigureS3.tif

**Supplementary Table**: brb371068‐sup‐0004‐TableS1.csv

**Supplementary Table**: brb371068‐sup‐0005‐TableS2.csv

**Supplementary Table**: brb371068‐sup‐0006‐TableS3.csv

**Supplementary Table**: brb371068‐sup‐0007‐TableS4.csv

**Supplementary Table**: brb371068‐sup‐0008‐TableS5.csv

**Supplementary Table**: brb371068‐sup‐0009‐TableS6.csv

**Supplementary Table**: brb371068‐sup‐0010‐TableS7.csv

## Data Availability

The data utilized in this study can be found within digital repositories on the internet, and specific information regarding the repository/repositories and access numbers is included in the supplementary material of the article.
