# Supplementary figures and images for: Genetic Liability to Bone Mineral Density and Functional Outcome After Ischemic Stroke
Source: Brain Behav. 2025 Nov 14;15(11):e71068. doi: 10.1002/brb3.71068 (PMC12617269; doi:10.1002/brb3.71068)

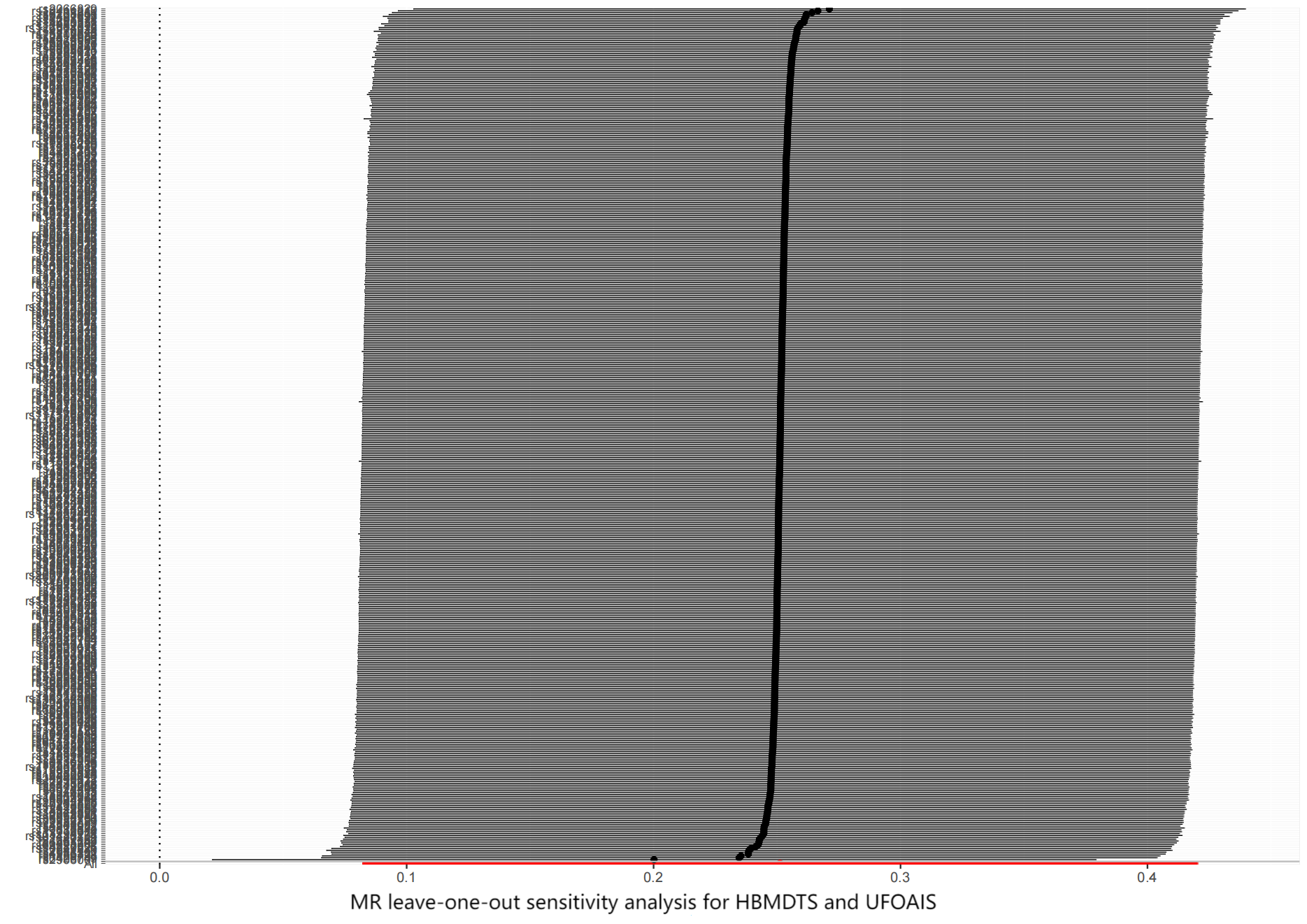

Supplement: Supplementary file 1 — Supplementary Figure: brb371068‐sup‐0001‐FigureS1.tif [file BRB3-15-e71068-s004.tif]

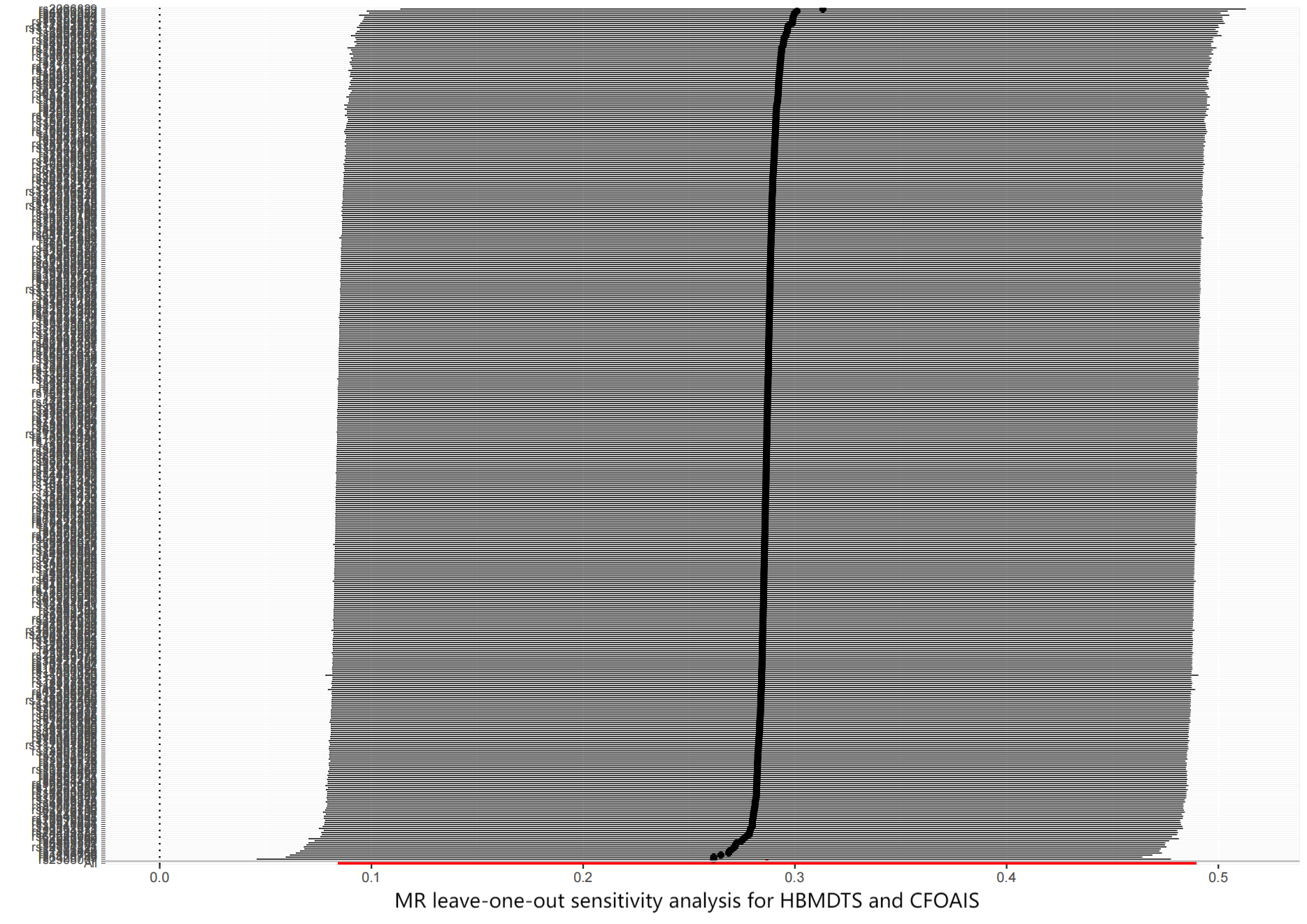

Supplement: Supplementary file 2 — Supplementary Figure: brb371068‐sup‐0002‐FigureS2.tif [file BRB3-15-e71068-s005.tif]

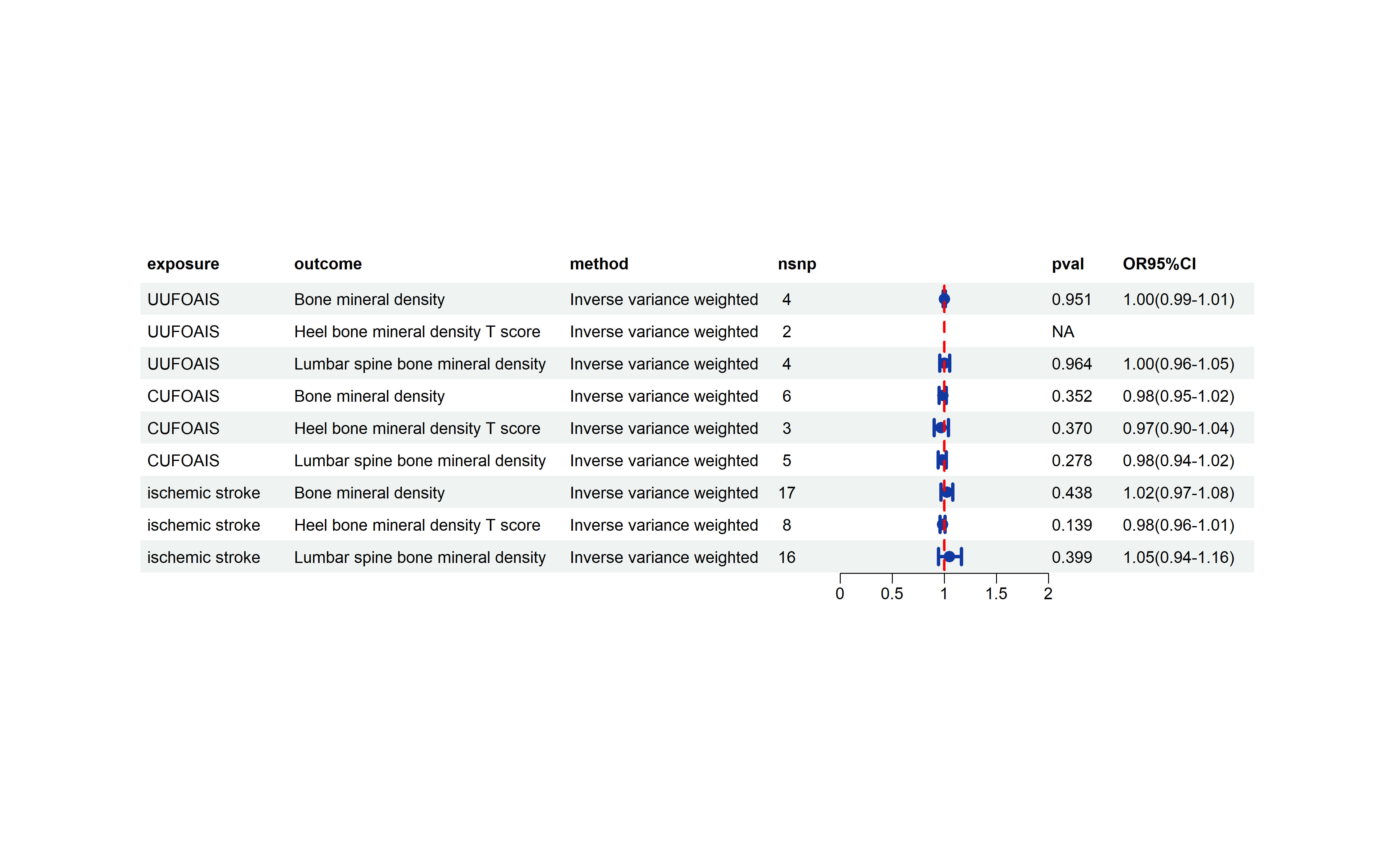

Supplement: Supplementary file 3 — Supplementary Figure: brb371068‐sup‐0003‐FigureS3.tif [file BRB3-15-e71068-s003.tif]
